# Developing and Testing a Smartphone Application to Enhance Adherence to Voice Therapy: A Pilot Study

**DOI:** 10.3390/ijerph20032436

**Published:** 2023-01-30

**Authors:** Vrushali Angadi, Ming-Yuan Chih, Joseph Stemple

**Affiliations:** 1 Department of Communication Sciences and Disorders, University of Kentucky College of Health Sciences, Lexington, KY 40536, USA; 2 Department of Health and Clinical Sciences, University of Kentucky College of Health Sciences, Lexington, KY 40536, USA

**Keywords:** voice therapy, voice disorders, adherence, mHealth, vocal function exercises

## Abstract

The present study aimed to develop a smartphone application (app) that addressed identified barriers to success in voice therapy; accessibility, and poor adherence to home practice. The study objectives were (1) to investigate if app use enhanced adherence to the home practice of voice therapy and (2) to test app usability. Maximizing the effectiveness of voice therapy is vital as voice disorders are detrimental to personal and professional quality of life. A single-blinded randomized clinical trial was completed for the first objective. Participants included normophonic individuals randomly assigned to the app group or the traditional group. The primary outcome measure was adherence measured as the number of missed home practice tasks. The second objective was completed through usability testing and a focus group discussion. The app group (*n* = 12) missed approximately 50% less home practice tasks as compared to the traditional group (*n* = 13) and these results were statistically significant (*p* = 0.04). Dropout rates were comparable between the two groups. Usability results were positive for good usability with high perceived usefulness and perceived ease of use. App use resulted in improved adherence to home practice tasks. App usability results were positive, and participants provided specific areas of improvement which are achievable. Areas for improvement include app engagement and willingness to pay.

## 1. Introduction

Maximizing the effectiveness of evidence-based voice therapies is the next step in the evolution of voice rehabilitation. With a lifetime prevalence of 29.9% [1] and a point prevalence of 6.6% [1] of the US population, voice disorders have a deleterious effect on both employment and social–emotional well-being. In the US teaching profession alone, the cost of lost workdays and voice treatment surpasses USD 2.5 billion annually (inflation applied) [2]. Voice disorders are prevalent in the head and neck cancer population, in individuals who use their voices occupationally, in children who misuse their voices, and in the growing population of older adults [3,4,5,6]. Outcomes research for voice therapy programs has demonstrated improved quality of life and overall vocal function in individuals with voice disorders [7,8]. However, research shows that 47% of patients who begin voice therapy do not complete the program, thus significantly reducing the effectiveness of treatment [9]. An established barrier to therapeutic success is poor adherence to voice therapy [9,10,11]. In an attempt to improve adherence to voice therapy, a study by Van Leer and Connor (2012) [9] demonstrated that the use of mobile-phone-based treatment videos increased adherence to voice therapy. Though not specific to voice therapy, previous studies have demonstrated that using mobile information and communication technologies, such as smartphones, to deliver evidence-based care (called mHealth) has been shown to improve patient adherence and clinically meaningful outcomes in fields, including addiction and diabetes [12,13,14]. Because of the increasing power, portability, and connectivity of mobile technologies, mHealth systems were able to provide a remote monitoring and assessment of patient treatment adherence and health status [15]. These conclusions were based on the system’s instant and personalized care that optimized and supported unique patient circumstances [16,17].

### Study Objective

The objective of this study was to investigate the influence of a smartphone-based app, MyVocalHealth^TM^ (MVH), on home practice adherence to an evidence-based voice therapy approach. The smartphone-based delivery method was compared to the standard of care which will be referred to as the traditional delivery method. We hypothesized that using a smartphone-based app delivery method would result in fewer missed home practice sessions, in comparison to the traditional delivery method. The voice therapy approach chosen was the Vocal Function Exercise (VFE) protocol. The reasons for choosing VFE were three-fold. (1) They are highly evidence-based with over 30 outcome studies showing reports of VFEs improving the normal voice, aging voice, trained voice and voice quality in the voice disordered population [8,18]. (2) Our study participants included non-treatment-seeking individuals with typical voices referred to as ‘normophonic’ in this paper. Previous studies in the literature have demonstrated that VFEs are capable of enhancing vocal capabilities in the normophonic voices [8]. (3) The VFE protocol is highly structured and prescriptive which makes it easy to be incorporated into an mHealth model. Since the smartphone app used in the study was newly developed, an assessment of its usability was also conducted and reported.

## 2. Materials and Methods

This study was designed as a pilot ‘proof-of-concept’ study and met the requirements for clinical trials specified by the National Institutes of Health [19]. The study was a randomized clinical trial (clinicaltrials.gov identifier: NCT04002336) and is subsequently reported per Consolidated Standards of Reporting Trials (CONSORT) guidelines for pilot and feasibility trials [20,21,22]. The study was conducted in accordance with the Declaration of Helsinki, and was approved by the Institutional Review Board at the University of Kentucky (protocol: 44183, approval date: 24 April 2018). Recruitment was completed between July and September 2018.

A total of 33 participants were recruited for the study and met the following inclusion criteria: adults (>18 years) with non-disordered/normophonic voice, non-smokers, hearing levels within functional limits, and iPhone users. Hearing within functional limits was defined as the participant’s ability to follow voice therapy instructions, imitate vocal exercises, and incorporate feedback provided during experimental tasks. iPhone users were defined as participants in possession of a mobile phone supported by iOS. The presence of vocal fold lesions or neurogenic voice disorders identified by endoscopic visualization and abnormal scores on auditory-perceptual evaluation constituted exclusion from the study. Additionally, participants with prior experience with Vocal Function Exercises were also excluded from the study. Participants who were non-treatment-seeking with normophonic voices were chosen, as this was the beta-testing phase of the smartphone app.

### 2.1. Study Protocol

Randomization: Following the completion of informed consent, thirty-three participants were randomized to one of two groups, i.e., the traditional (*n* = 16) group or the app (*n* = 17) group. A 1:1 blocked randomization sequence, using blocks of 2, 3, and 4, was determined for group assignment. Informed consent was completed by all study participants.

Assessment protocol: Baseline data collection for both groups included the maximum phonation time (MPT) (in seconds) and the maximum airflow volume (MAV) (in ml). The MAV was determined using the KAYPENTAX Phonatory Aerodynamic System 6600 (Pentax Medical, Montvale, NJ, USA) and was used to determine an individual MPT goal for each participant. Each participant’s MAV was divided by a standard airflow rate of 80 mL/s; 80 mL/s approximates the lower end of the standard airflow rate (70–200 mL/s) described by Hirano [23]. This method helps to individualize MPT goals and was recommended by Stemple and Hapner (2019) for use when completing the VFE protocol [18]. Additionally, the app group participated in a three-step usability evaluation, as listed in the next paragraph. Post-intervention data collection for both groups included MPT calculations. Participants from the app group completed an additional questionnaire related to their experiences with app use. Following study completion, six participants from the app group were randomly chosen to participate in a focus group to provide feedback on app use.

Usability evaluation: A three-step mixed-method approach was employed to evaluate the usability of the app. First, a usability testing session was conducted at the beginning of the participant’s first study visit. The purpose was to understand their initial impression and the challenges of using the app. During testing, participants were asked to complete the first VFE exercise on the app by following a voice-prompted scenario. The participants were asked to share their thoughts by using the “think-aloud” protocol while they interacted with the app [24]. The testing session was video-recorded for later analysis. Secondly, the participants completed a usability survey at the end of the trial. Lastly, six app group participants were randomly selected for a focus group. The purpose of the focus group was to solicit their experiences and suggestions after using this app for a longer duration of the study period. In the focus group, participants first shared their experiences of using this app, such as the timing and locations of their app use. Following descriptions of their user experience, participants offered and rated their improvement ideas for this app. The focus group data were recorded by the facilitator on flip charts during the focus group.

**Figure 1:**
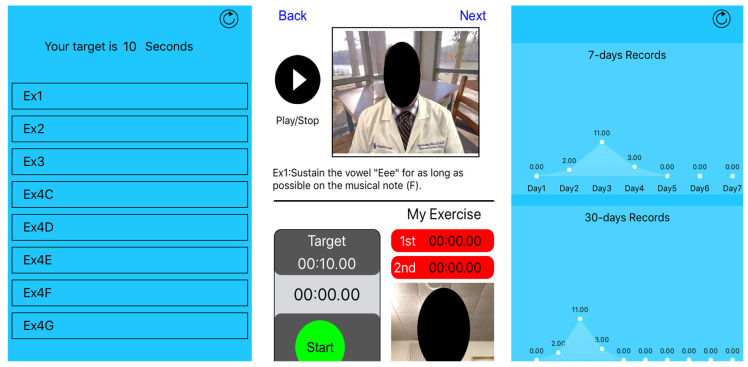
MyVocalHealth^TM^ (MVH) interface for Vocal Function Exercises.

App features: The app was provided at no cost and app access was discontinued following study completion. The app consisted of customized sets of VFE videos. Participants were assigned the video set which matched their gender and vocal range. The VFE program consists of four exercises. The fourth exercise, divided into five tasks, was performed on certain musical notes which matched with the participant’s habitual vocal range. The first exercise and all the fourth exercise tasks require the user to maximally sustain each note and document the time in seconds. This is referred to as the maximum phonation time (MPT). In this application, the user/participant starts a clinician-modeled video of each VFE task. Clinician modeling is vital as the VFEs are performed on a specific vocal posture known as the semi-occluded vocal tract [25]. As the user begins the sustained phonation task, they are prompted to start a timer built into the app. Starting the timer triggers the phone camera to video-record the practice session. Above the timer, the clinician-prescribed MPT goal is provided to cue the patient about how long the note should be sustained. The participant stops the timer after each task, which also terminates the video recording. The user is then prompted to review the recorded exercise video and/or upload the video. If satisfied with the attempt, the user can choose to upload the video or redo the exercise. Only the attempts reviewed and approved by the participant are uploaded for the researcher to view. However, to avoid multiple attempts from participants, the ‘dosage’ of exercise attempts for VFEs was reviewed and reiterated during every follow-up session, i.e., each exercise should be performed two times each, twice a day. These instructions were reiterated to discourage the participants from applying multiple attempts in search of the “best time” or “best attempt”. The user’s attempt is automatically sent to a Health Insurance Portability and Accountability Act (HIPAA)-secure medical server, monitored by the treating clinician. The patient logs are registered in a web-based clinician portal via a web-browser on their computer to view the user’s exercise date and time, maximum sustained phonation, and video recordings. Consequently, the app allows for non-patient-dependent adherence reporting and monitoring. Figure 1 shows the current app interface including the start page, exercise task page, and weekly MPT graph.

Intervention: Following baseline data collection, participants in both groups met with a research assistant to learn Vocal Function Exercises (VFEs), as described by Stemple (1994) [26]. The research assistants (RAs) were four undergraduate students studying Communication Sciences and Disorders. Two research assistants had no prior experience with the administration of VFEs and two research assistants had previously helped with a study that required them to administer VFEs. To ensure the accuracy and consistency of the technique, the first author trained RAs in VFEs through individual and group sessions. Additionally, the first author monitored 20% of all RA-conducted sessions with study participants. The study lasted six weeks and participants were required to attend weekly sessions of voice therapy with the first author and RAs. Participants were monetarily compensated for their participation.

Outcome measures: The primary outcome measure was adherence to the home practice of voice therapy, as measured by the number of missed home practice voice therapy ‘tasks’. Each exercise and its attempt constituted a ‘task’. The VFE protocol consists of four exercises and each exercise is completed twice (*n* = 16 tasks) [18]. The VFE protocol is to be completed two times per day. As a result, the participant was expected to complete a total of 32 exercise tasks per day [18]. The secondary outcome measure was the percentage of MPT goal achieved, following the completion of the 6-week VFE program. The traditional group was provided with home practice log sheets and an audio file of a clinician completing VFEs. This method was adopted to mimic a realistic clinic situation where patients were taught exercises, given home practice log sheets, and provided with an audio file of VFEs to help with home practice. The traditional group was requested to be honest with logging in home practice attempts. Both groups were informed that missed sessions could negatively affect their post-intervention MPT. The app group allowed for automatic logging in home practice sessions. Please refer to the section on app features for a more detailed explanation.

Usability assessment measures: The usability survey included the System Usability Scale (SUS), the Health Information Technology Usability Evaluation Survey (Health-ITUES), and the user version of the Mobile Application Rating Scale (uMARS) [27]. A ten-item SUS instrument was used to assess the perceived usability with high internal consistency (Cronbach’s alpha = 0.83–0.97) [28]. The Health-ITUES is a 20-item customizable scale measuring four constructs of IT usability: the quality of work life (i.e., the impact of the app on treatment and recovery), perceived usefulness, the perceived ease of use, and user control (Cronbach’s alpha = 0.81–0.95) [29]. The uMARS was designed to assess the usability of a mobile app in app quality (e.g., engagement and aesthetics), subject quality items (e.g., willingness to recommend or pay), and the perceived impact on health behaviors (Cronbach’s alpha = 0.7–0.9) [30].

**Figure 2:**
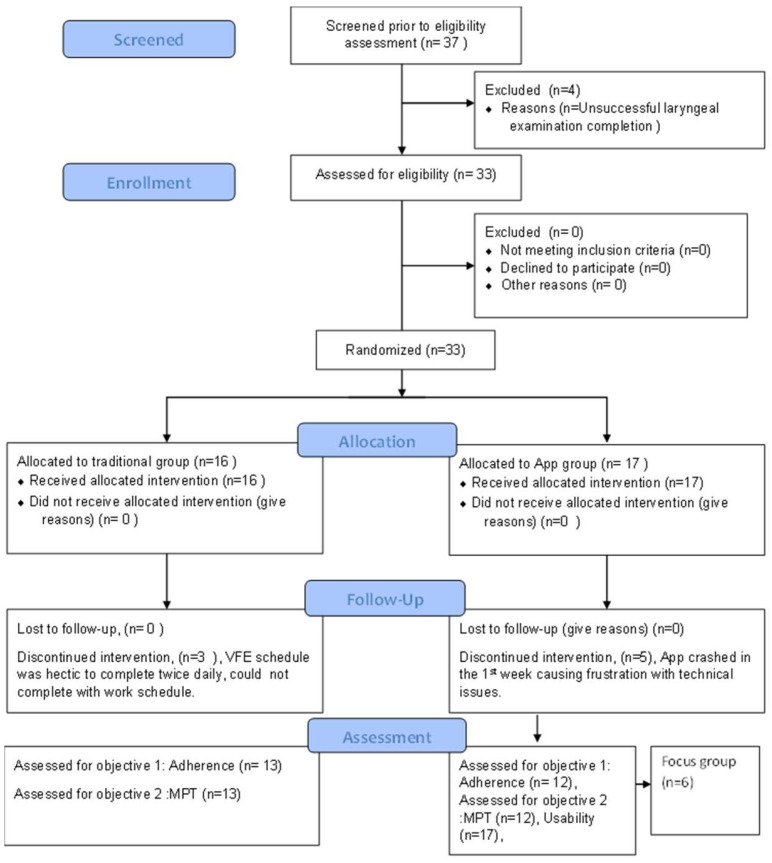
CONSORT flow chart of enrollment, allocation, follow-up, and analysis [20,21,22].

Blinding: Participants were blinded to the study objective and knowledge of the primary outcome measure, i.e., adherence. Participants were informed that the focus of the study was to compare the effectiveness of two delivery methods of voice therapy, as measured by the post-intervention MPT. The participants were not blinded for the group assignment because they either received or did not receive the app. Figure 2 shows the CONSORT flow chart detailing the enrollment, allocation, follow-up, and analysis of study participants [20,21,22].

### 2.2. Statistical Analyses

Data analyses for primary, secondary, and usability outcome measures were completed using SPSS (ver. 25, IBM, SPSS Inc., Chicago, IL, USA). Normality tests were administered prior to completing comparison statistics. The appropriate parametric (independent *t*-test) or non-parametric test (Mann–Whitney U) was applied based on the normality of distribution. The adherence data were not normally distributed and consequently a non-parametric test (Mann–Whitney U) was applied for comparisons. The MPT data were normally distributed and, consequently, a parametric test (*t*-test) was used for comparisons. Significance values were set at *p* ≤ 0.05. Descriptive statistics were provided for usability measurements.

Qualitative Usability Evaluation. The video recordings in the initial scenario-based usability test were coded by a research assistant who was not part of usability testing to minimize bias. An investigator who was present at the testing reviewed 20% of the coding to confirm its accuracy. The participant’s app interaction in this test was broken down into nine steps, which include the following: (1) open the app; (2) open the first VFE exercise; (3) play the exercise video; (4) stop the exercise video; (5) start recoding; (6) stop recording; (7) review the recorded video; (8) complete the first VFE exercise for the second time; and (9) open the second VFE exercise. These nine steps are necessary user interactions or experiences with the app in order to complete the scenario described earlier. If a participant completed all nine steps successfully, the testing completion rate was considered as 100%. The descriptive statistics of the testing completion rate were reported. Each user’s unique comments recorded during the ‘think aloud’ process were coded for each participant. These comments/statements were representative of the user’s interactions with the app. For example, if a user commented that this app was easy to use more than once, we only coded this user comment as “app easy to use” once for this participant. A summary of the comments made by the participants during the scenario testing and the post-study focus group were used for analysis.

## 3. Results

For ease of discussion, the results section is divided into results from the clinical trial and usability evaluation.

### 3.1. Clinical Trial

Twenty-five participants (F = 22, M = 3, age range: 18–23 years) completed the study. A total of eight participants opted out of the study. Five participants from the app group opted out of citing issues with app use. The app crashed for users following usage on multiple devices. This version was soon repaired/modified by the second author and no technical issues were encountered for the remainder of the study. Three participants from the traditional group cited difficulty completing exercise tasks on a regular basis.

Primary Outcomes: Data analysis for the primary outcome measure of adherence demonstrated a total of 202 ((Standard deviation/SD): 79) missed home practice sessions in the app group and 409 ((Standard deviation/SD): 274) sessions in the traditional group. The difference in adherence to home practice was statistically significant between groups (0.041). Both groups demonstrated improvements in the MPT for the secondary outcome measure of the percent MPT goal achieved. The two groups were not significantly different (*p* = 0.73) for the secondary outcome measure. Table 1 shows adherence and MPT comparisons between the two groups.

### 3.2. Usability Evaluation

Initial Usability Testing: A total of 17 participants completed the initial scenario-based usability test. The mean task completion rate exceeded 95%. The most frequently missing steps were playing and stopping the tutorial videos (four out of these five participants). The analysis of the think-aloud data during testing showed that most participants felt the app was easy to use (*n* = 13, 72%) and that the example VFE videos were useful (7, 39%). However, eight (44%) participants identified some interface issues, including the following: “the Next button is confusing (*n* = 4)”, “don’t know whether to do exercise with the video or after (*n* = 4)”, “confusing to use (*n* = 1)”, and “video could be on one screen and then do the exercise in the next screen (*n* = 1)”.

Usability Survey: Twelve app group participants completed the usability survey at the post-test. Overall, the app showed good usability with a mean SUS score of 78.13 (see Table 2), which is a B+ rating according to the Sauro–Lewis curved grading scale [31]. A mean uMARS app quality score of 3.7 was obtained, which differs from an acceptable threshold value of 3 [27]. Mean scores of greater than 4 (Likert scale: 1–5) were obtained and were representative of high perceived usefulness, perceived ease of use, and information design. However, the app can be improved in user engagement ratings with a mean score lower than 2. The scores obtained in the engagement subscale were clearly indicative of the need to improve customization; specifically the ability to customize the settings and preferences (e.g., sound, content, and notifications, M = 1.83, SD = 1.27)) and the interactivity (i.e., the ability to facilitate user input, provide feedback, and contain prompts (reminders, sharing options, notifications, etc.), M = 2.08, SD = 1.16)). Table 2 shows a summary of survey results from usability testing scales.

Posttest Survey: The 12 app group participants who completed the post-test surveys also shared brief open-ended comments with us about how the app was helpful. A majority of participants (10, 77%) commented that the app helped them practice the VFE and improved their vocal function or voice quality. However, one participant preferred in-person sessions over using the VFE app and two participants did not leave comments.

Focus group: Six app group participants joined a focus group to provide further information about their experiences. The three primary locations of app use were their homes, cars (especially at the parking lot), and restaurants. The majority followed the recommendation to conduct their daily VFE sessions once in the morning and the other in the evening. However, some completed VFE sessions during their lunch break. Several improvement ideas were identified. The top five were: (1) adding a clear indication for exercise competition; (2) adding a customizable reminder and snoozing function; and (3) showing their best VFE records on top. These improvement ideas were consistent with weaknesses identified in the usability surveys about customization and interactivity. The new version of the app has addressed these issues.

## 4. Discussion

The purpose of this study was to determine if smartphone-app-aided voice therapy delivery improved adherence to the home practice of voice therapy tasks compared to the traditional delivery method. Additionally, usability testing was completed through surveys, “think aloud” methods, and focus-group discussions.

A single-blinded randomized clinical trial was completed with normophonic participants (*n* = 33). Participants were randomly assigned to the traditional group or app group and were blinded to the primary outcome measure, i.e., adherence to home practice tasks. The secondary outcome measure was maximum phonation time (MPT). Results from adherence comparisons are in favor of app-enhanced delivery as the app group demonstrated increased adherence to home practice tasks as compared to the traditional group, and these results were statistically significant (*p* = 0.041). The MPTs increased in both groups, demonstrating the effectiveness of voice therapy, but were comparable and differences were not statistically significant. Usability analyses of the app from surveys were echoed in focus group discussions where participants reported easy app usability and interfaces. A systematic quantitative and qualitative analysis approach was used based on reports in previous studies, especially those focused on device or app development. Using a combined approach allowed the investigative team to paint a holistic picture of app capabilities.

Decisions on the components included in the app interface during the development phase were based on the important work completed by Van Leer and colleagues in the area of investigating the drivers and barriers that influence adherence to voice therapy [10]. Although many important themes emerged from Van Leer et al.’s study, it was unrealistic to address all components in the app prototype. Consequently, we addressed themes conducive to patient success within the realm of the availability and expertise in technology development. The themes from Van Leer’s (2010) study included the complexity of voice exercises, the fear of completing exercises inaccurately in the absence of a clinician model, and the lack of motivation. A follow-up study by Van Leer and colleagues (2012) assessed the impact of using MP4 portable digital players with examples of therapy videos on patient adherence and motivation. According to the authors, the study demonstrated a “modest but statistically significant positive effect of MP4 videos therapy examples on practice frequency and motivation when compared to standard of care” [9]. Given these study findings, the app prototype, MyVocalHealth^TM^ (MVH), includes clinician videos describing and modeling each exercise, a graph depicting the trajectory of MPT gains, and the ability for home practice videos to be accessible to the clinician instantly for feedback. The study team hypothesized that clinician videos would aid with visual feedback and enhance accuracy; graphing MPT gains would improve motivation and the ability to receive periodic clinician feedback would address the issue of accuracy with home practice. The current study differed from the Van Leer study (2012) in terms of key components [9], including the voice therapy intervention (Vocal Function Exercises), adherence monitoring, and device capabilities. The present study employed Vocal Function Exercises that have a strong evidence base [8], offered non-patient dependent adherence monitoring with greater accuracy or reporting abilities, and used the participant’s own smartphone device which offered greater familiarity with device use. From a clinician desirability stand-point, this was the only app at the time of development that allowed non-patient-dependent adherence monitoring and the automation of data collection and entry (MPTs). In 2021, Van Leer and colleagues also published results of the iOS-based voice therapy app which offered non-patient dependent adherence monitoring [11].

Besides ease of use, it is important to address the other intent behind the app which was to develop a modality to improve accessibility to specialty services such as voice therapy. A smartphone-based platform was purposefully chosen based on national statistics. In 2017, a survey from the Pew research center (PRC) demonstrated that 77% of the US population owned a smartphone. This percentage increased to 97% in 2021 [32]. The survey also showed that a greater percentage of the population owned a smartphone as compared to the tablet or desktop computers. Additionally, PRC reports also show an increased percentage of smartphone use versus desktop/tablet computers in rural areas [33] and by individuals with disabilities [34]. Therefore, a smartphone-based platform has the potential to alleviate accessibility issues resulting from geographic and individual disparities.

## 5. Limitations

The present study is not without limitations. First, issues encountered the following app dissemination possibly that affected patient attrition rates. The app crashed during the first week of the study and participants reported difficulties with uploading videos. We believe that this event led to the higher dropout rate seen in the app group as compared to the traditional group as the greatest proportion of participants dropped out in the first week. There is no doubt that this event possibly led to greater frustration related to app use even with the existing participants and possibly influenced motivation levels. Second, we were limited to iOS-based smartphones as the app only works with the iOS interface. Resultantly, we missed valuable feedback from users of the Android platform. Third, we did not directly address patient motivation, as in the studies by Van Leer and colleagues. We can make indirect inferences based on usability results; however, we would be extrapolating. Fourth, although available through the app, study participants were not provided with immediate feedback to ensure that all participants received the same degree of attention from the clinician. Possibly, this decision led to the outcome of comparable MPTs in both groups. However, comparable outcomes are also representative of the non-inferiority of app-enhanced voice therapy delivery when compared to the standard of care. This is an important finding for a proof-of concept study. Fifth, the study participants were non-treatment-seeking normophonic individuals. As much as we attempted to replicate real-world settings, the absence of a voice disorder with no negative implications on quality of life has the potential to influence patient motivation to adhere to an intervention. Finally, the app includes only one voice intervention, Vocal Function Exercises. The VFE program is not a ‘one size fits all’ program and can limit the dissemination reach of the app.

## 6. Future Directions

The current study is the first step aimed to develop and enhance technology that improves patient outcomes through improved accessibility and breaking down barriers that affect therapeutic success. We received valuable feedback from our participants which will be incorporated into the next version of the app. Additionally, data collection is underway for a clinical trial investigating app-enhanced voice therapy and its effect on adherence and drop-out rates in the treatment-seeking clinical population. As we develop the app further, we hope to incorporate more evidence-based voice therapy interventions. It is important to acknowledge the success of other evidence-based voice therapy programs and include them in future versions of the app to ensure that the chosen voice intervention is tailored to patient needs. 

## 7. Conclusions and Implications for Practice

This study was completed before the COVID-19 pandemic. In addition to the points stated above, the current global pandemic has grossly magnified the issue of lack of accessibility to healthcare services. Unfortunately, this lack of accessibility to healthcare has long been part of an ongoing discussion. The pandemic has pushed medical and rehabilitation services to improve access and one of the first steps was giving providers the ability to bill for telehealth services. The smartphone-based platform could positively enhance already-existing telehealth services by offering voice therapy at one’s finger tips and remote patient monitoring. In January 2022, the American Speech–Language and Hearing Association (ASHA) introduced a new family of codes for remote patient monitoring. According to the ASHA, “CMS agreed that these services are important to beneficiaries and will allow therapists—including SLPs (speech-language pathologists)—and certain other nonphysician providers to bill the Remote Therapeutic Monitoring (RTM) codes, as written”. CMS is the center for Medicare and Medicaid Services [35]. This could potentially result in greater interest for centers to procure the app to ensure reimbursement.

## Figures and Tables

**Table 1:** Missed home practice tasks (adherence) and maximum phonation time percentage comparisons between the traditional and app groups.

Group	*n*	Missed Home Practice (Means and SD)	*p*-Value (Mann–Whitney U-test)	MPT (Means and SD Percentage of 80% MPT Goal)	*p*-Value (*t*-test)
Traditional	13	409 (274)	0.041 *	89.9 (16.8)	0.729
App	12	202 (79)		89.54 (15.9)	

(Significance: *p* ≤ 0.05 *, SD = Standard Deviation).

**Table 2:** Descriptive statistics of usability measures (SD = Standard Deviation).

Usability Measures	Mean	Median	SD	Min.	Max.
System Usability Scale (SUS)					
Overall Usability	78.13	81.25	16.21	52.5	97.5
Health IT Usability Evaluation Scale (Health-ITUES)					
Quality of Work Life	3.91	4	0.9	2.33	5
Perceived Usefulness	4.22	4.11	0.73	2.89	5
Perceived Ease of Use	4.46	4.6	0.51	3.6	5
User Control	3.42	3.5	0.71	1.67	4.67
User Version of the Mobile Application Rating Scale (uMARS)					
uMARS-App Quality					
Engagement	2.95	2.8	0.78	1.4	4.6
Functionality	3.77	3.87	1.18	1.25	5
Aesthetics	3.94	4.17	0.84	2.67	5
Information	4.15	4.38	0.91	2.25	5
Overall Quality	3.7	3.74	0.83	2.27	4.84
uMARS-Subjective Quality					
Recommend	3.91	4	1.16	2	5
Future Use	3.67	4	1.15	1	5
Willing to Pay	2.33	2	1.15	1	4
Start Rating	3.55	4	0.82	2	5
uMARS-Perceived Impact					
Overall Impact	3.93	4	1.03	2.33	5

## Data Availability

The raw data for the study are unavailable. The data are not reported to a third party. The clinicaltrials.gov site has information regarding the study, but data are not available.

## References

[1] Roy N., Merrill R.M., Gray S.D., Smith E.M. (2005). Voice disorders in the general population: Prevalence, risk factors, and occupational impact. Laryngoscope.

[2] Verdolini K., Ramig L.O. (2001). Review: Occupational risks for voice problems. Logop. Phoniatr. Vocol..

[3] Hseu A.F., Spencer G., Woodnorth G., Kagan S., Kawai K., Nuss R.C. (2021). Barriers to voice therapy in dysphonic children. J. Voice.

[4] Angadi V., Dressler E., Stemple J. (2017). A multidimensional study of vocal function following radiation therapy for laryngeal cancers. Ann. Otol. Rhinol. Laryngol..

[5] Hapner E.R. (2017). The changing landscape of vocal needs in the aging baby boomer. Perspect. ASHA Spec. Interest Groups.

[6] Etter N.M., Stemple J.C., Howell D.M. (2013). Defining the lived experience of older adults with voice disorders. J. Voice.

[7] Speyer R. (2008). Effects of voice therapy: A systematic review. J. Voice.

[8] Angadi V., Croake D., Stemple J. (2019). Effects of Vocal Function Exercises: A Systematic Review. J. Voice.

[9] van Leer E., Connor N.P. (2012). Use of portable digital media players increases patient motivation and practice in voice therapy. J. Voice.

[10] van Leer E., Connor N.P. (2010). Patient perceptions of voice therapy adherence. J. Voice.

[11] van Leer E., Lewis B., Porcaro N. (2021). Effect of an iOS app on voice therapy adherence and motivation. Am. J. Speech-Lang. Pathol..

[12] Quanbeck A., Chih M.-Y., Isham A., Johnson R., Gustafson D. (2014). Mobile delivery of treatment for alcohol use disorders: A review of the literature. Alcohol Res. Curr. Rev..

[13] Kleinman N.J., Shah A., Shah S., Phatak S., Viswanathan V. (2017). Improved Medication Adherence and Frequency of Blood Glucose Self-Testing using an m-Health Platform Versus Usual Care in a Multisite Randomized Clinical Trial Among People with Type 2 Diabetes in India. Telemed. e-Health.

[14] Yoo W., Shah D.V., Chih M.Y., Gustafson D.H. (2020). A smartphone-based support group for alcoholism: Effects of giving and receiving emotional support on coping self-efficacy and risky drinking. Health Inform. J..

[15] Chih M.Y., McCowan A., Whittaker S., Krakow M., Ahern D.K., Aronoff-Spencer E., Hesse B.W., Mullett T.W., Vanderpool R.C. (2020). The Landscape of Connected Cancer Symptom Management in Rural America: A Narrative Review of Opportunities for Launching Connected Health Interventions. J. Appalach. Health.

[16] Chih M.-Y., Patton T., McTavish F.M., Isham A.J., Judkins-Fisher C.L., Atwood A.K., Gustafson D.H. (2014). Predictive modeling of addiction lapses in a mobile health application. J. Subst. Abus. Treat..

[17] Nahum-Shani I., Smith S.N., Spring B.J., Collins L.M., Witkiewitz K., Tewari A., Murphy S.A. (2016). Just-in-Time Adaptive Interventions (JITAIs) in mobile health: Key components and design principles for ongoing health behavior support. Ann. Behav. Med..

[18] Stemple J.C., Hapner E.R. (2019). Voice Therapy: Clinical Case Studies.

[19] NIH’s Definition of a Clinical Trial. https://grants.nih.gov/policy/clinical-trials/definition.htm.

[20] Rennie D. (2001). CONSORT revised—Improving the reporting of randomized trials. JAMA.

[21] Piaggio G., Elbourne D.R., Pocock S.J., Evans S.J., Altman D.G. (2012). Reporting of noninferiority and equivalence randomized trials: Extension of the CONSORT 2010 statement. JAMA.

[22] Eldridge S.M., Chan C.L., Campbell M.J., Bond C.M., Hopewell S., Thabane L., Lancaster G.A. (2016). CONSORT 2010 statement: Extension to randomised pilot and feasibility trials. Pilot Feasibility Stud..

[23] Hirano M. (1981). Clinical examination of voice. Disord. Hum. Commun..

[24] Broekhuis M., van Velsen L., Hermens H. (2019). Assessing usability of eHealth technology: A comparison of usability benchmarking instruments. Int. J. Med. Inform..

[25] Titze I.R. (2006). Voice training and therapy with a semi-occluded vocal tract: Rationale and scientific underpinnings. J. Speech Lang. Hear. Res..

[26] Stemple J.C., Lee L., D’Amico B., Pickup B. (1994). Efficacy of vocal function exercises as a method of improving voice production. J. Voice.

[27] Adam A., Hellig J.C., Perera M., Bolton D., Lawrentschuk N. (2018). ‘Prostate Cancer Risk Calculator’ mobile applications (Apps): A systematic review and scoring using the validated user version of the Mobile Application Rating Scale (uMARS). World J. Urol..

[28] Lewis J.R. (2018). The system usability scale: Past, present, and future. Int. J. Hum. –Comput. Interact..

[29] Yen P.Y., Wantland D., Bakken S. Development of a Customizable Health IT Usability Evaluation Scale. Proceedings of the AMIA Annual Symposium Proceedings.

[30] Stoyanov S.R., Hides L., Kavanagh D.J., Wilson H. (2016). Development and Validation of the User Version of the Mobile Application Rating Scale (uMARS). JMIR Mhealth Uhealth.

[31] Sauro J., Lewis J.R. (2016). Quantifying the User Experience: Practical Statistics for User Research.

[32] Perrin A. (2021). Mobile technology and Home Broadband 2021. https://www.pewresearch.org/internet/wp-content/uploads/sites/9/2021/06/PI_2021.06.03_Mobile-Broadband_FINAL.pdf.

[33] Vogels E. (2021). Some Digital Divides Persist between Rural, Urban and Suburban America. https://policycommons.net/artifacts/1808201/some-digital-divides-persist-between-rural-urban-and-suburban-america/2543052/.

[34] Perrin A., Atske S. (2021). Americans with Disabilities Less Likely than Those without to Own Some Digital Devices. https://www.pewresearch.org/fact-tank/2021/09/10/americans-with-disabilities-less-likely-than-those-without-to-own-some-digital-devices/.

[35] ASHA 2022 Medicare Part B Final Rule Includes New Remote Monitoring Codes, Significant Payment Cuts. https://www.asha.org/news/2021/2022-medicare-part-b-final-rule-includes-new-remote-monitoring-codes-significant-payment-cuts/.

